# Shaping future practices: German-speaking medical and dental students’ perceptions of artificial intelligence in healthcare

**DOI:** 10.1186/s12909-024-05826-z

**Published:** 2024-08-06

**Authors:** Sebastian Fitzek, Kyung-Eun Anna Choi

**Affiliations:** 1 Health Services Research, Faculty of Medicine/Dentistry, Danube Private University, Steiner Landstraße 124, Krems‑Stein, 3500 Austria; 2 Center for Health Services Research, Brandenburg Medical School, Seebad 82/83, 15562 Rüdersdorf b. Berlin, Neuruppin, Germany

**Keywords:** Artificial intelligence, Healthcare, Medical education, Survey, Perceptions, Digital literacy, Students

## Abstract

**Background:**

The growing use of artificial intelligence (AI) in healthcare necessitates understanding the perspectives of future practitioners. This study investigated the perceptions of German-speaking medical and dental students regarding the role of artificial intelligence (AI) in their future practices.

**Methods:**

A 28-item survey adapted from the AI in Healthcare Education Questionnaire (AIHEQ) and the Medical Student’s Attitude Toward AI in Medicine (MSATAIM) scale was administered to students in Austria, Germany, and Switzerland from April to July 2023. Participants were recruited through targeted advertisements on Facebook and Instagram and were required to be proficient in German and enrolled in medical or dental programs. The data analysis included descriptive statistics, correlations, t tests, and thematic analysis of the open-ended responses.

**Results:**

Of the 409 valid responses (mean age = 23.13 years), only 18.2% of the participants reported receiving formal training in AI. Significant positive correlations were found between self-reported tech-savviness and AI familiarity (*r* = 0.67) and between confidence in finding reliable AI information and positive attitudes toward AI (*r* = 0.72). While no significant difference in AI familiarity was found between medical and dental students, dental students exhibited slightly more positive attitudes toward the integration of AI into their future practices.

**Conclusion:**

This study underscores the need for comprehensive AI education in medical and dental curricula to address knowledge gaps and prepare future healthcare professionals for the ethical and effective integration of AI in practice.

**Supplementary Information:**

The online version contains supplementary material available at 10.1186/s12909-024-05826-z.

## Introduction

Artificial intelligence (AI) is rapidly transforming healthcare, with promising applications developing in several specialties, such as radiology, pathology, dermatology, and dentistry [29]. The challenges imposed by artificial intelligence require significant adaptation in medical education to ensure readiness [2]. The ability of AI to enhance the accuracy of medical imaging, streamline surgical procedures, and improve diagnostic capabilities demonstrates its potential to significantly improve patient care [4, 10]. AI's potential in medicine is vast and varied [15].

However, the effective integration of AI into clinical practice requires more than technological innovation alone. Healthcare professionals who are prepared, knowledgeable, and receptive to the potential of AI are also needed. Recent research underscores the importance of integrating AI education into undergraduate medical and dental curricula to equip future physicians and dentists with the knowledge and skills necessary to work effectively in a healthcare environment that is increasingly reliant on AI [24, 30].

Although researchers widely acknowledge the importance of AI literacy in healthcare [11, 22], the educational needs and perspectives of medical and dental students regarding AI, particularly within different regional and cultural contexts, have not been fully explored. Comprehensive AI policy education frameworks are essential for university teaching and learning [7]. AI deployment in healthcare faces numerous challenges that need to be addressed [13]. Culture and language can influence how individuals perceive and interact with new technologies, making it crucial to adapt educational approaches to meet the diverse needs of learners in various settings. Moreover, the variation in teaching and learning approaches across medical and dental education programs necessitates individualized AI curricula that address regional variations in challenges and opportunities [12].

To address this gap, we focused our research on the perceptions of, knowledge about, and training experiences with AI among German-speaking medical and dental students in Austria, Germany, and Switzerland. These countries share a common language and cultural heritage while also having distinct healthcare systems and educational approaches. By examining perspectives on AI within this specific context, we aim to inform the development of tailored educational interventions and policy recommendations that effectively address the unique challenges and opportunities faced by these countries. Trends indicate a growing need for AI literacy in medical curricula [16].

We drew upon established learning theories, such as constructivism and social learning theory, to guide our investigation. Constructivism emphasizes the active role learners play in constructing their knowledge, while social learning theory underscores the importance of social interaction and observation in the learning process. These theories suggest that AI education should be learner-centered, interactive, and relevant to the specific context of medical practice [9, 18].

Our overarching research question is as follows: What are the perceptions of German-speaking medical and dental students regarding the integration of AI into their future professional practices? Through this inquiry, we aim to:

1. The gaps in AI literacy among medical and dental students were identified.
2. To develop evidence-based educational strategies that align with established learning theories.
3. The preparedness of future healthcare professionals for the ethical and effective utilization of AI in their careers should be enhanced.

## Materials and methods

### Study design and participants

A cross-sectional survey was conducted from April to July 2023 among medical and dental students in Germany, Austria, and Switzerland. Participants were recruited through targeted advertisements on Facebook and Instagram, focusing on individuals within the specified age range, geographic locations, and fields of study. The eligibility criteria included current enrollment in a medical or dental program and proficiency in the German language.

### Survey instrument

The survey instrument was adapted from a prior study by Bisdas et al. [5], incorporating elements from the AI in Healthcare Education Questionnaire (AIHEQ, [20]) and the Medical Student’s Attitude Toward AI in Medicine (MSATAIM) scale [23]. The survey contained 28 items covering sociodemographic data, understanding of AI principles, AI in medical education, and attitudes toward AI. The survey was translated into German by a bilingual expert and refined through cognitive interviews with native German-speaking medical students. Improving the quality of web surveys is critical for accurate data collection [14]. Content validity was assessed by a panel of experts in artificial intelligence and medical education, with feedback quantified using the content validity ratio (CVR) and content validity index (CVI) metrics. The instrument’s internal consistency (reliability) was assessed using Cronbach’s alpha (α = 0.807).

### Data collection

The survey was administered online using Google Forms. Before starting, participants provided electronic consent and confirmed their eligibility (age over 18 and current enrollment in a medical or dental program). Anonymity was ensured throughout the data collection process.

### Statistical analysis

We used G*Power to conduct an a priori power analysis, determining the required sample size to detect a medium effect size (f2 = 0.15) for multiple linear regression with three predictors (tech-savviness, AI familiarity, and confidence in finding reliable AI information), given a power of 0.80 and an alpha level of 0.05. This effect size was chosen based on both a preliminary assessment of our data and the literature in the field of AI and medical education ([5] reported an effect size of 0.14 for a similar analysis [20], observed a range of effect sizes from 0.10 to 0.18 in their study). Data normality was assessed using the Shapiro‒Wilk test and Q‒Q plots, informing the selection of appropriate parametric or nonparametric tests. Descriptive statistics (means, ranges, standard deviations), t tests, correlations (Pearson’s r), and multiple linear regression analyses were performed using Python 3.10. Analyses focused on examining relationships between variables, including differences in AI perspectives between students with and without formal AI training, and evaluating predictors of attitudes toward AI.

### Qualitative data analysis

The open-ended responses were analyzed via thematic analysis. Initial open coding extracted keywords and concepts, which were then grouped into themes and subthemes based on recurring patterns and relationships. Multiple researchers independently reviewed the data to ensure intercoder reliability, with any discrepancies resolved through discussion and consensus. Atlas.ti software (version 8) was used to facilitate the coding process.

### Ethics approval

The study received ethical approval from the Ethics Committee of Danube Private University.

## Results

### Demographic characteristics

The study participants were primarily young adults (mean age = 23.13 years, SD = 4.27), with a balanced gender distribution (49.6% men, 45.5% women, 2.9% nonbinary, and 2.0% unspecified). Most were medical (57.0%) or dental (43.0%) students enrolled in various stages of their education, providing diverse perspectives on AI. The majority were in the preclinical/bachelor stage (59.2%), followed by those in the clinical/master stage (33.0%), and a smaller proportion were pursuing a doctorate/Ph.D. (7.8%). Geographically, most respondents were from Germany (45.5%), followed by Austria (34.7%) and Switzerland (19.8%).

### Familiarity with AI applications and tech savviness

**Fig. 1:**
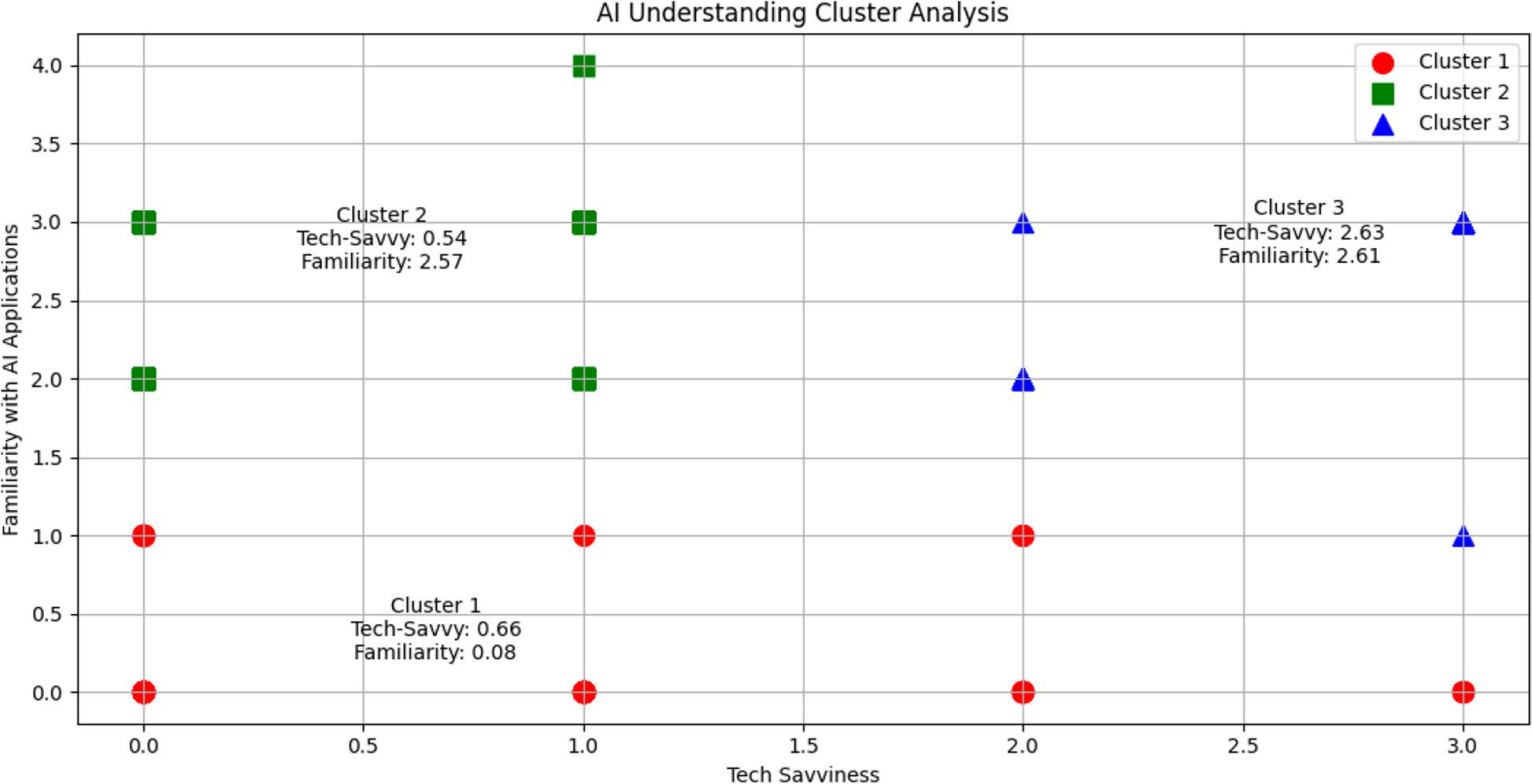
Cluster analysis of tech savviness and AI familiarity

A significant proportion of respondents (52.3%) reported no familiarity with AI applications, while only a small percentage (4.4%) indicated being very familiar. Self-assessed tech savviness varied considerably, with no significant association found between tech affinity and familiarity with AI applications in medicine. Cluster analysis identified three distinct groups based on tech-savviness and AI familiarity: basic users, intermediate users, and proficient users (Fig. 1).

### AI understanding by gender and age

**Fig. 2:**
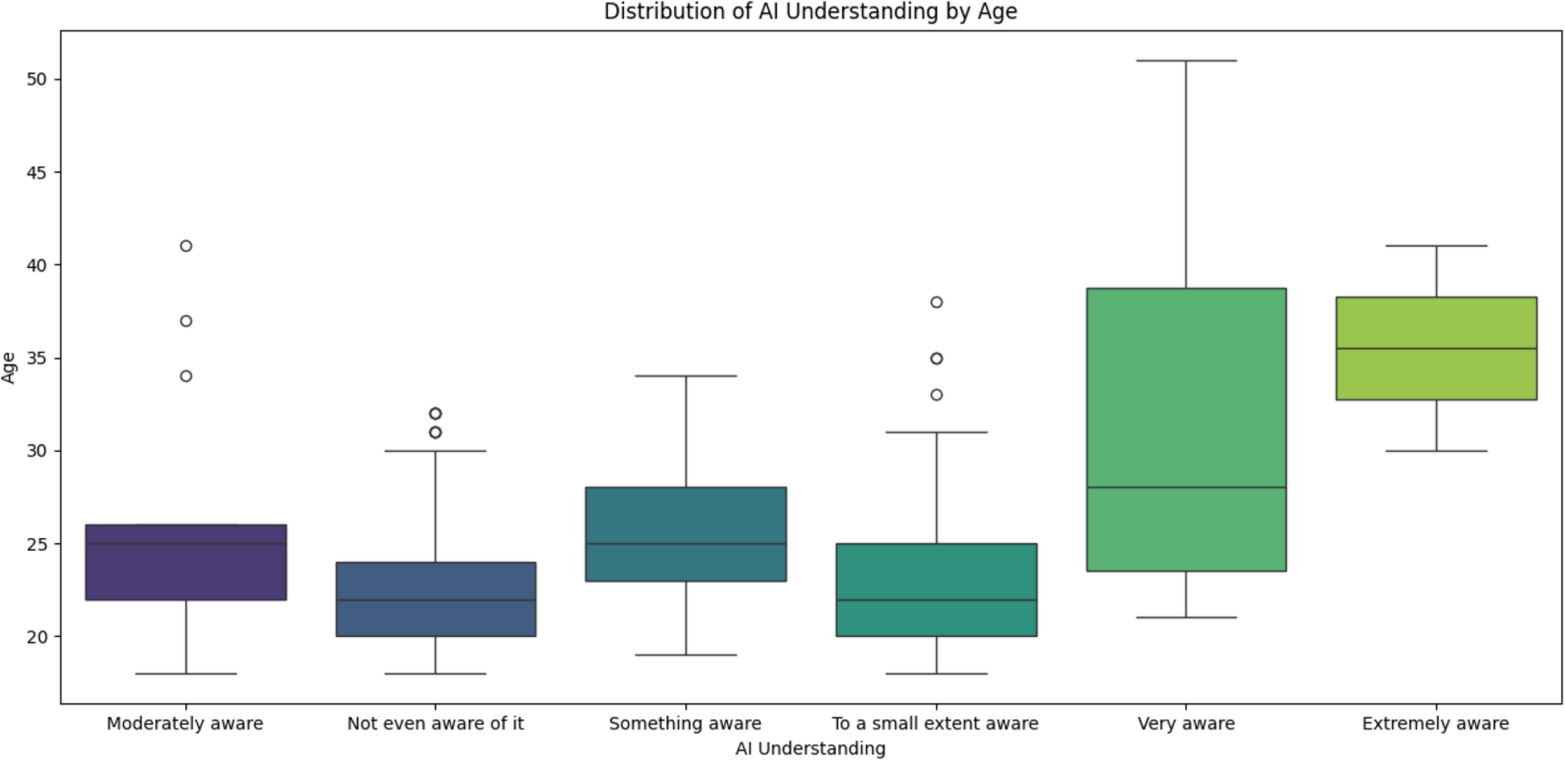
How AI understanding varies with age

**Fig. 3:**
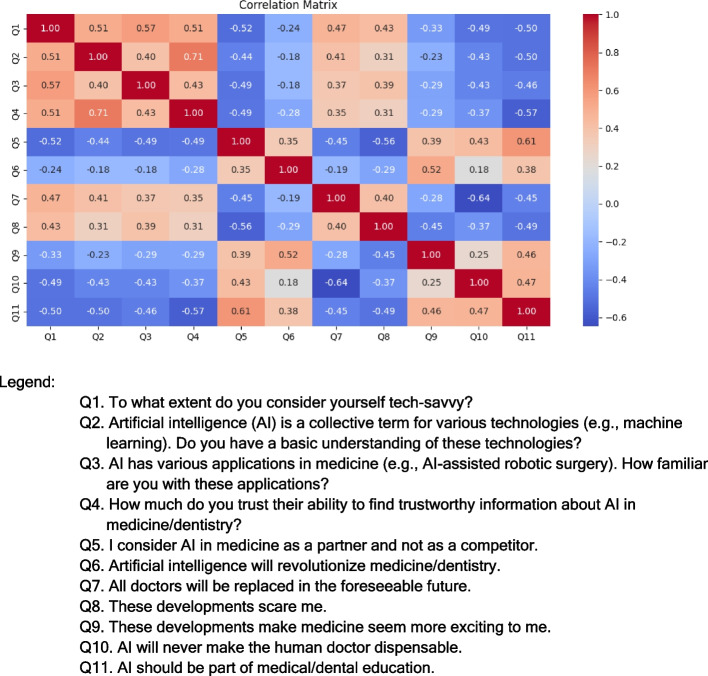
Correlation matrix for technological proficiency levels and familiarity with AI applications. Legend: Q1. To what extent do you consider yourself tech-savvy? Q2. Artificial intelligence (AI) is a collective term for various technologies (e.g., machine learning). Do you have a basic understanding of these technologies? Q3. AI has various applications in medicine (e.g., AI-assisted robotic surgery). How familiar are you with these applications? Q4. How much do you trust their ability to find trustworthy information about AI in medicine/dentistry? Q5. I consider AI in medicine as a partner and not as a competitor. Q6. Artificial intelligence will revolutionize medicine/dentistry. Q7. All doctors will be replaced in the foreseeable future. Q8. These developments scare me. Q9. These developments make medicine seem more exciting to me. Q10. AI will never make the human doctor dispensable. Q11. AI should be part of medical/dental education

Analysis of AI understanding by gender (Figure A18) revealed a diverse range of comprehension across all gender identities, with no one group demonstrating significantly greater familiarity with AI. Age was associated with AI understanding, with younger participants generally reporting lower levels of AI comprehension than older participants (Fig. 2). There was no significant difference in AI understanding between medical and dental students across age groups or gender identities (Fig. 3).

### Attitudes toward AI

Table 1 summarizes respondents’ views on the adoption of AI in healthcare, assessed on a scale from 0 to 5, where higher scores indicate greater agreement.

The respondents strongly agreed that AI has the potential to revolutionize healthcare and improve medical care. Opinions were divided on whether AI would replace all doctors, with some expressing concerns. While fear of AI development was not widespread, a minority expressed strong concerns. Excitement about AI’s potential to advance medical practice was prevalent, and there was strong support for incorporating AI into medical education.

**Table 1:** Attitudes toward AI

Variable	Mean	SD	Min	25th Pctl	Median	75th Pctl	Max
AI revolutionizing medicine/dentistry	4.76	0.67	1	5	5	5	5
All doctors being replaced in the future	2.14	1.13	1	1	3	3	5
Fear of AI developments	1.76	1.31	1	1	1	3	5
Excitement about AI developments	4.64	0.82	1	5	5	5	5
AI making doctors dispensable	3.73	1.13	1	3	3	5	5
AI improving medicine	4.74	0.67	3	5	5	5	5
AI as part of medical education	4.17	1.04	1	3	5	5	5

### Correlation analysis

Correlation analysis revealed significant positive associations between attitudes favoring AI’s potential to revolutionize medicine and the belief that AI will improve medical practices (*r* = 0.61). Conversely, negative correlations emerged between positive attitudes toward AI and the belief that AI could replace human doctors.

### Comparison between medical and dental students

No significant difference was found in AI familiarity between medical (mean = 2.76, SD = 0.89) and dental students (mean = 2.85, SD = 0.85; *p* = 0.123). However, dental students demonstrated slightly more positive attitudes toward AI’s potential to revolutionize medicine (medical students: mean = 4.72, SD = 0.68; dental students: mean = 4.81, SD = 0.65; *p* = 0.032). This suggests that dental students might perceive AI as having more direct or immediate applications in their field, potentially due to the increasing use of AI in diagnostic and treatment planning tools specific to dentistry.

### Qualitative data from open-ended questions

Qualitative analysis of the open-ended responses identified three key themes:

1. Need for foundational AI instruction
2. Desire to integrate AI into existing curricula
3. Concerns about the potential dehumanization of care

These findings underscore the need for a balanced approach to AI adoption in medical education that incorporates both technical skills and ethical considerations.

## Discussion

This study investigated German-speaking medical and dental students’ attitudes and perceptions toward AI, revealing a diverse range of understanding. Our findings underscore the urgent need for customized AI educational approaches within medical and dental programs to address variability in knowledge and technical proficiency. AI familiarity and attitudes varied significantly across educational stages, with tech-savviness increasing as students progressed in their studies (*F*=54.93, *p*<0.0001). AI systems can predict patient deterioration in emergency settings [25]. This highlights the necessity of adaptable educational strategies throughout medical training.

Despite a generally positive sentiment toward AI, a significant gap in formal AI education exists, with only 18.2% of students receiving structured training. This underscores the need for comprehensive AI curricula, as advocated in previous research [5, 23]. Primary care will see significant changes due to AI integration [6]. A significant positive correlation (*r*=0.72) was found between students’ confidence in sourcing reliable AI information and their favorable attitudes toward AI. This strong association suggests that enhancing technological competence and providing accessible, trustworthy AI resources could be pivotal in fostering more receptive attitudes toward AI in medical practice [8].

Our findings align with global discussions on AI education in medical fields, confirming the need for specific policies and ethical instructional design. AI educational programs using AI can significantly impact learning outcomes [17]. Similar to observations in Canada, the UK, and the US, our study identifies prevalent gaps in formal AI instruction, highlighting a common international challenge in integrating AI into medical education [3, 21, 26]. These findings hold particular significance in the German-speaking European context, enriching our understanding of medical student AI perceptions and contributing to the broader discourse on AI in medical informatics.

The significant positive correlation between confidence in sourcing reliable AI information and favorable attitudes toward AI suggests that both knowledge and the quality and accessibility of information are crucial. The emerging field of mobile health integrates AI technologies [27]. An ethical instructional design should ensure equitable access to AI knowledge, address potential biases in AI algorithms, and teach critical appraisal skills for AI information. Practical implementations include incorporating case studies on AI ethics, providing resources on bias in AI, and ensuring diverse representations in AI training materials. AI's role in democracy and healthcare is critical [19].

Our findings revealed significant variations in AI understanding across gender categories, aligning with other studies (e.g., [28]) and challenging assumptions about gender disparities in technological proficiency within the medical field. This emphasizes the need for gender-inclusive AI education policies to create a workforce that is both inclusive and prepared for AI, ensuring that individuals across all demographics have fair access to AI knowledge and skills.

Furthermore, our study adds a new dimension by exploring the specific context of German-speaking medical and dental students. While previous research has focused primarily on other regions, our findings highlight that the need for comprehensive AI education is a global phenomenon, emphasizing the importance of tailoring AI education to specific cultural and linguistic contexts.

Potential limitations in our study design should be acknowledged, particularly regarding the potential for selection bias introduced by recruiting participants through social media platforms. This approach may have led to an overrepresentation of tech-savvy individuals, affecting the generalizability of our findings, as those who were more comfortable with technology might have been more likely to participate in the survey. Although we assessed participants’ self-reported technology use in the survey, this might not fully capture the nuances of technological proficiency. Therefore, the findings may not be completely generalizable to the broader population of German-speaking medical and dental students. Future studies should consider using a more diverse range of recruitment methods to enhance representativeness.

Additionally, although we took measures to mitigate common method bias (e.g., careful formulation of survey questions, ensuring participant anonymity, and employing a mixed-methods approach), the use of a single survey instrument for data collection may have introduced some bias. However, Harman’s single-factor test did not suggest that common method bias was a major concern in this study.

Efforts were made to address nonresponse bias by keeping the survey short and engaging and by sending follow-up reminders to maximize participation. We compared the demographic characteristics of our sample (e.g., age, sex, year of study) with available data on the broader population of German-speaking medical and dental students to assess representativeness. However, despite these efforts, the potential for bias remains, and future research should aim to verify these findings in more diverse and representative samples.

### Limitations and future directions

This study has limitations. The cross-sectional design and self-reported data may not fully capture evolving attitudes toward AI or provide a comprehensive assessment of AI knowledge. Additionally, the sampling method may introduce selection bias. Future research should prioritize longitudinal studies and objective measures, such as assessments of AI knowledge or skills using standardized tests or simulations, to track changes in AI perceptions and education needs over time.

## Conclusion

This study provides compelling evidence for significant variations in AI familiarity and attitudes among German-speaking medical and dental students, underscoring the urgent need for comprehensive and adaptable AI education programs tailored to different educational stages and cultural contexts. Medical school curricula must adapt to the digital age [31]. Enhancing technological competence and providing access to reliable AI information are crucial for fostering more receptive attitudes toward AI in healthcare.

The study’s findings challenge assumptions about gender disparities in technological proficiency and emphasize the need for inclusive AI education policies. By addressing the identified gaps and implementing inclusive, comprehensive AI education programs, we can empower future healthcare professionals to confidently and ethically navigate the AI-driven landscape of modern medicine, ultimately leading to improved patient care and a more equitable healthcare system.

### Supplementary Information

Supplementary Material 1.

## Data Availability

The datasets generated and/or analyzed during the current study are not publicly available because the data are anonymous and voluntary survey responses. However, they are available from the corresponding author upon reasonable request.

## Abbreviations

AI: Artificial intelligence
DL: Deep learning
ML: Machine learning
SD: Standard deviation
NAN: No answer
