# Supplementary material for: Shaping future practices: German-speaking medical and dental students’ perceptions of artificial intelligence in healthcare
Source: BMC Med Educ. 2024 Aug 6;24:844. doi: 10.1186/s12909-024-05826-z (PMC11304766; doi:10.1186/s12909-024-05826-z)
Supplement: Supplementary file 1 — Supplementary Material 1. [file 12909_2024_5826_MOESM1_ESM.docx]

**Appendix**

 Supplementary Table 1: Socio-demographics of study sample

| **Category** | **Details** |
| --- | --- |
| Age | Mean: 23.13 years, Range: 18-51, SD=4.27 yrs |
| Gender | Male: 203, Female: 186, Non-binary: 12, Not specified: 8 |
| Field of Study | Human Medicine: 233 (56.97%), Dentistry: 176 (43.03%) |
| Study Phase | Pre-clinic/Bachelor: 242 (59.17%), Clinic/Master: 135 (33.01%), Doctorate/PhD: 32 (7.82%) |
| Study Year | 1st: 99, 2nd: 116, 3rd: 79, 4th: 35, 5th: 42, 6th: 38 |
| Country | Germany: 186, Austria: 142, Switzerland: 81 |
| Prior AI Training | No basic understanding: 214, basic understanding to a small extent: 135, Some basic understanding: 42, Very knowledgeable: 18 |

A.1: Age Distribution


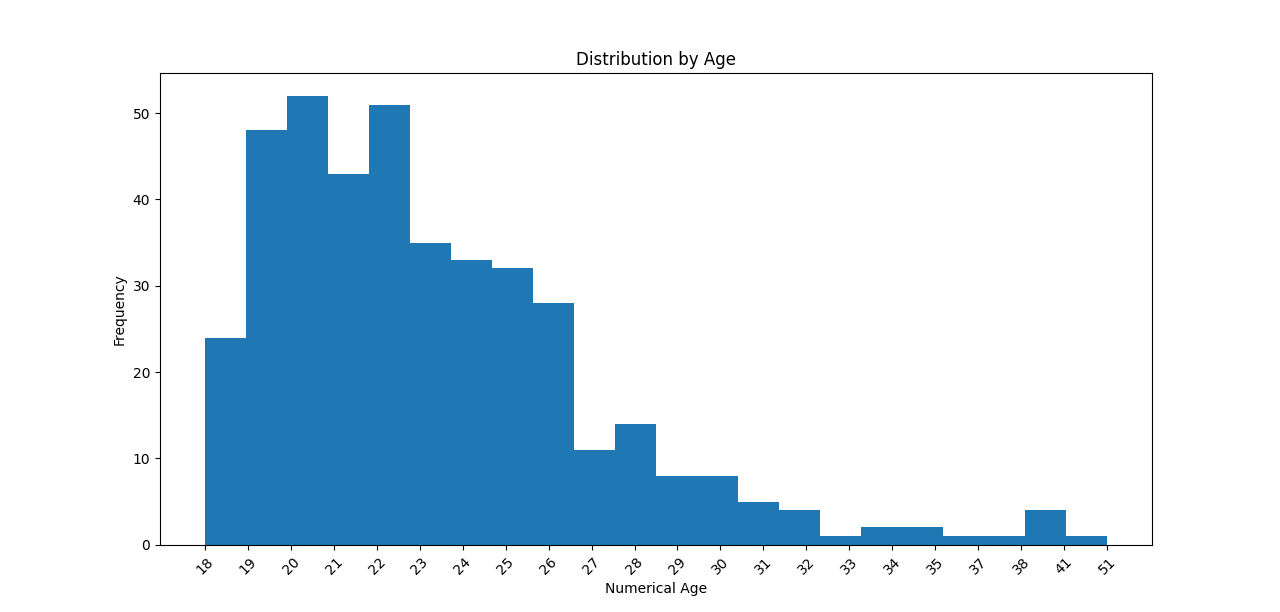


A.2: Gender Distribution


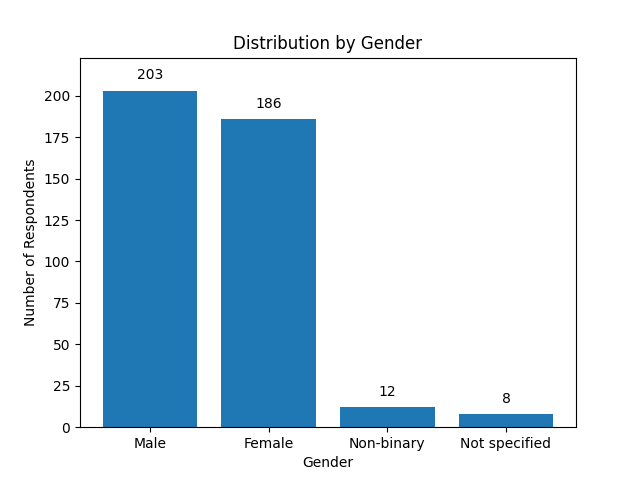


A.3: Distribution by Study Phase^[[1]](#footnote-1)^

**
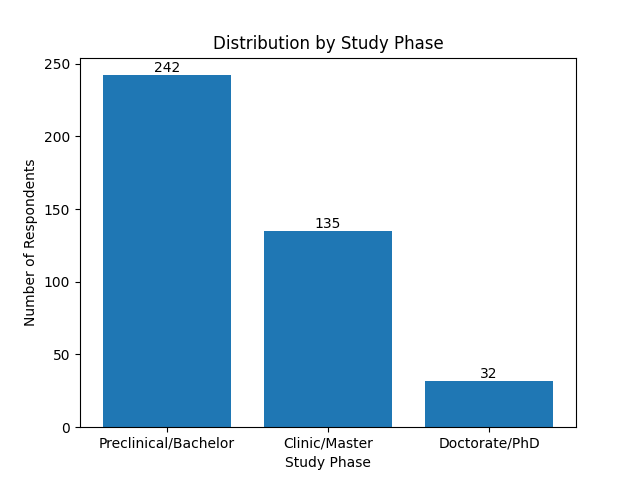
**

A.4: Distribution by Current Study Year


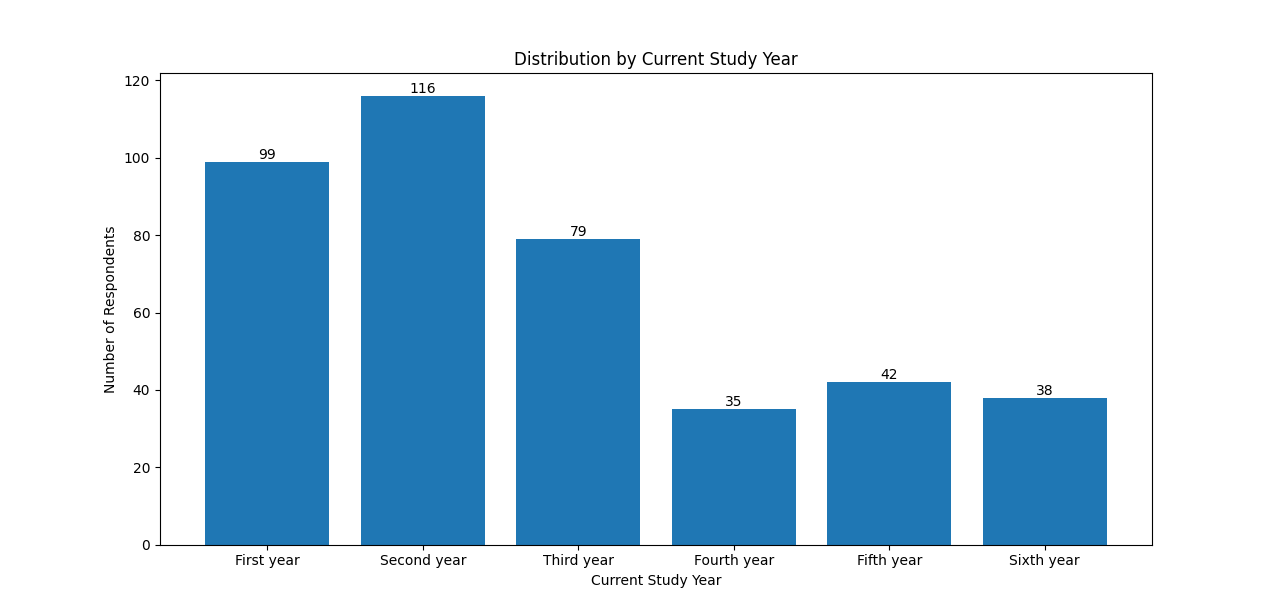


A.5: Distribution by Country


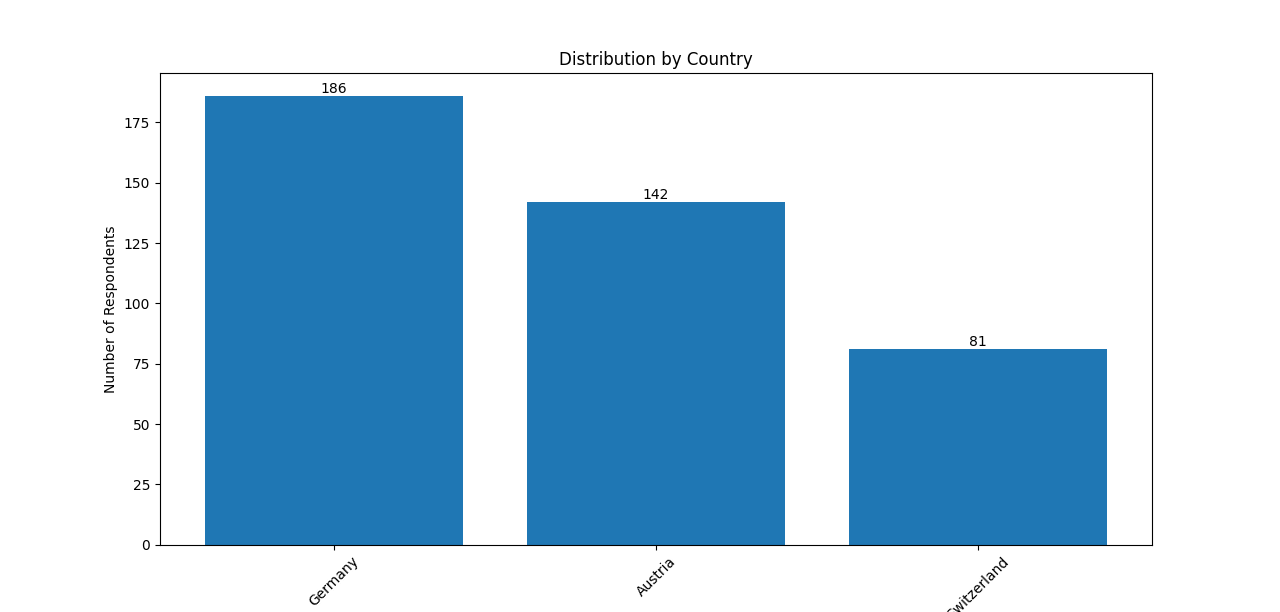


A.6: Prior AI Training in Medical/Dental Education


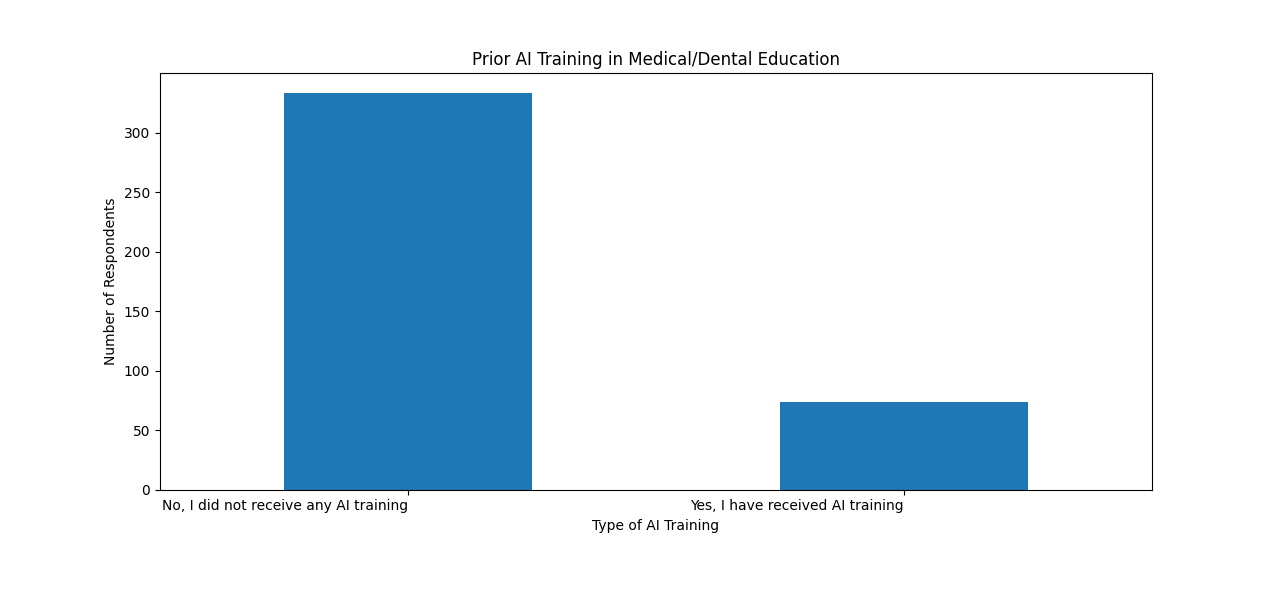


#### A.7: Distribution of Perception of AI as a Partner Not Competitor

*
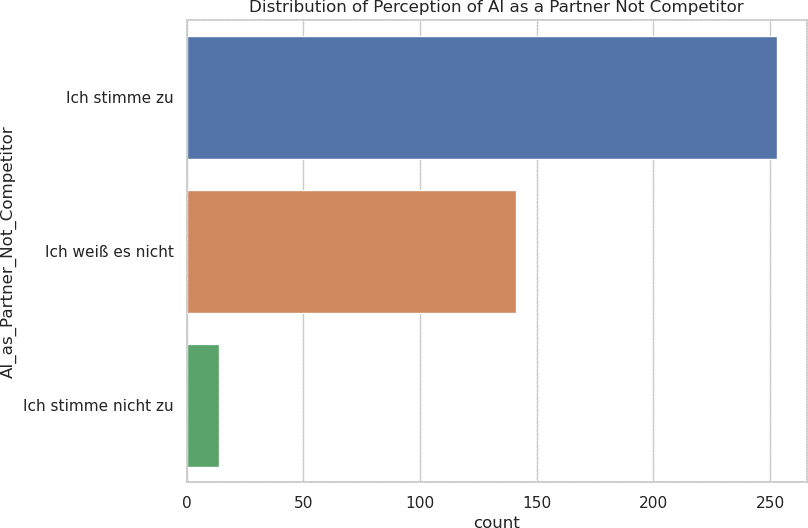
*

I do not agree know

I don’t know

I agree

A8: Opinion on AI’s Potential to Revolutionize Medicine


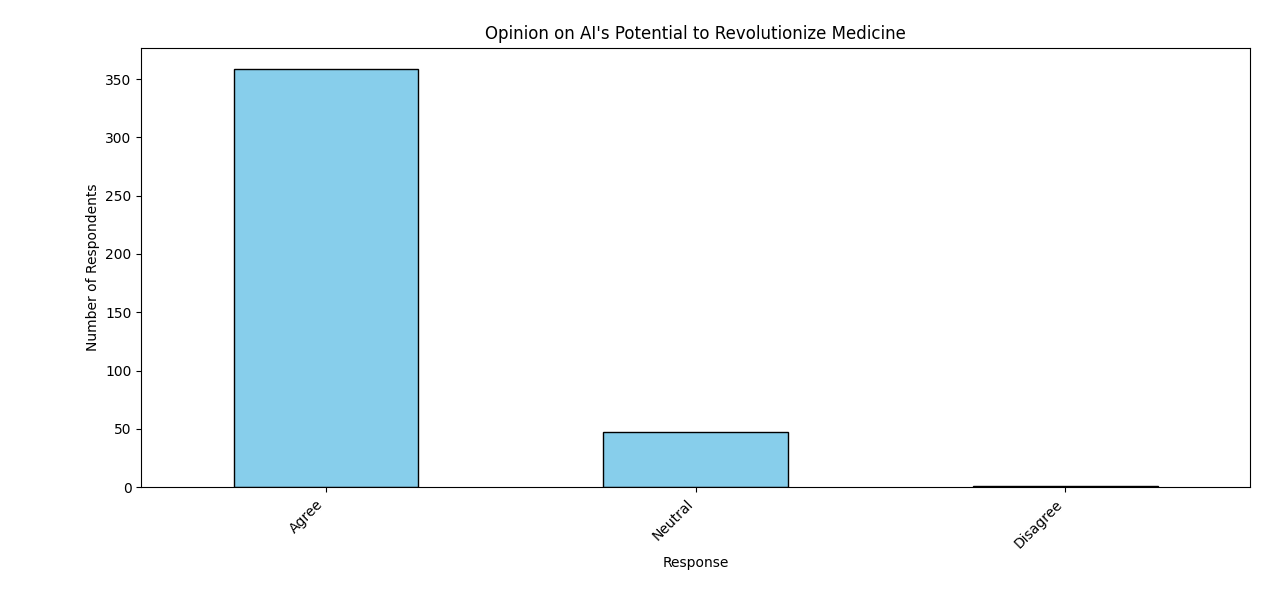


A9: Artificial Intelligence (AI) is a collective term for many technologies (e.g. ML). Do you have a basic undestanding of these tehnologies?


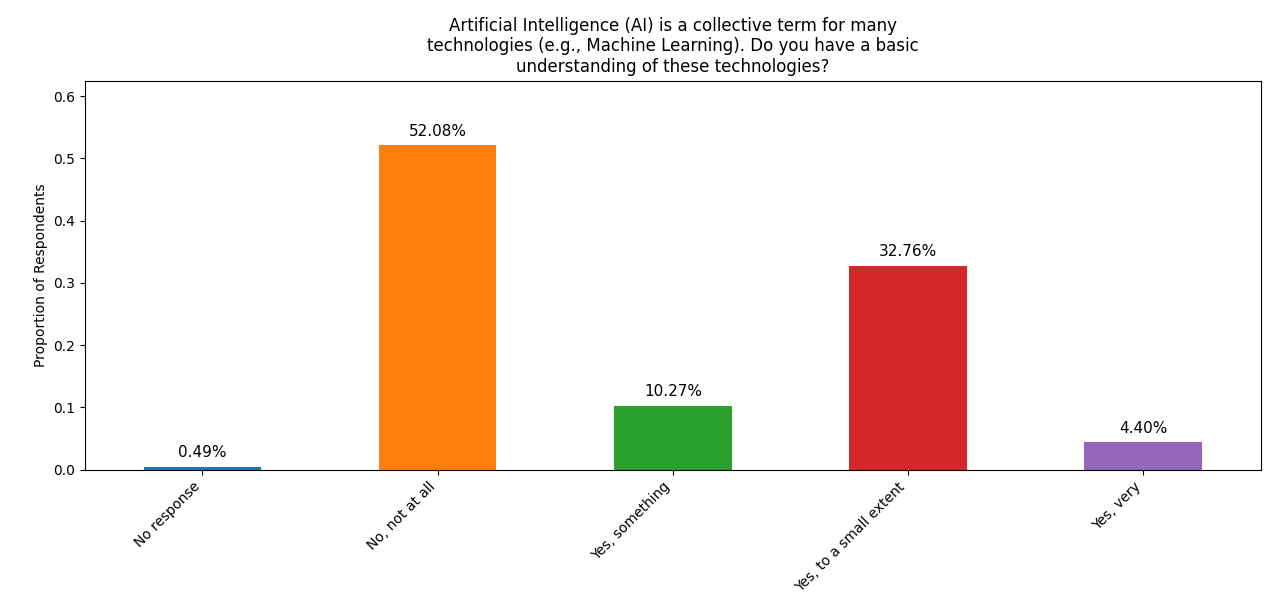


A10: AI has many applications in medicine (e.g., AI-assisted robotic surgery). How familiar are you with there applications?


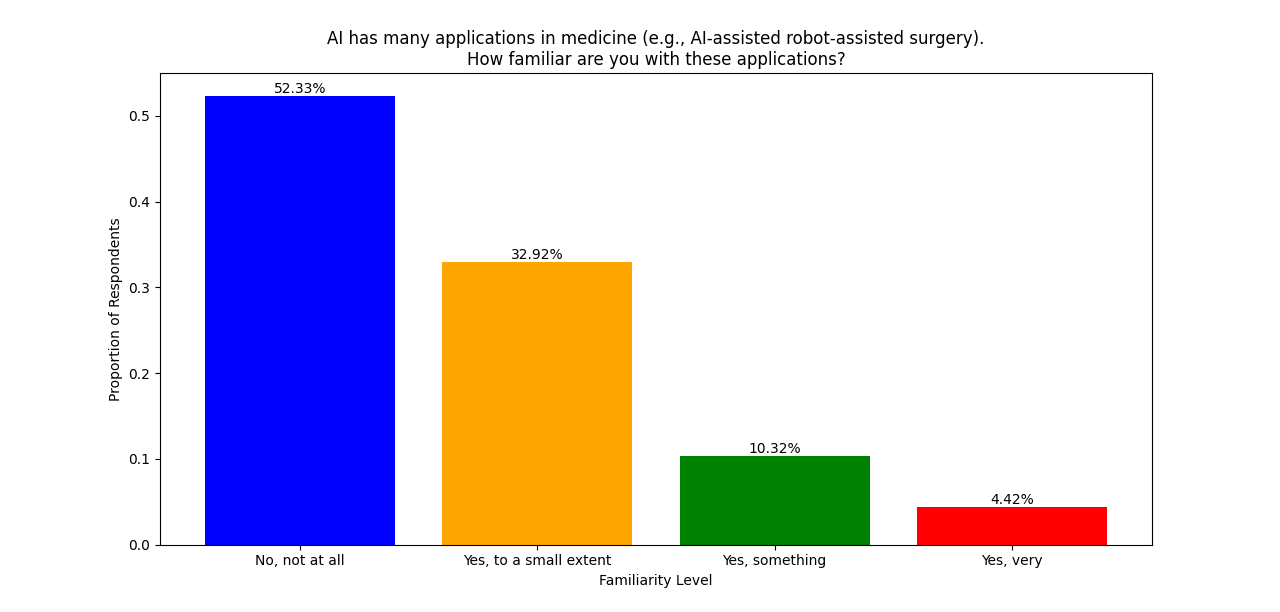


A11: Many applications we use in daily life already use AI (e.g., speech/text recognition, email spam filter). How familiar are you with these applications?


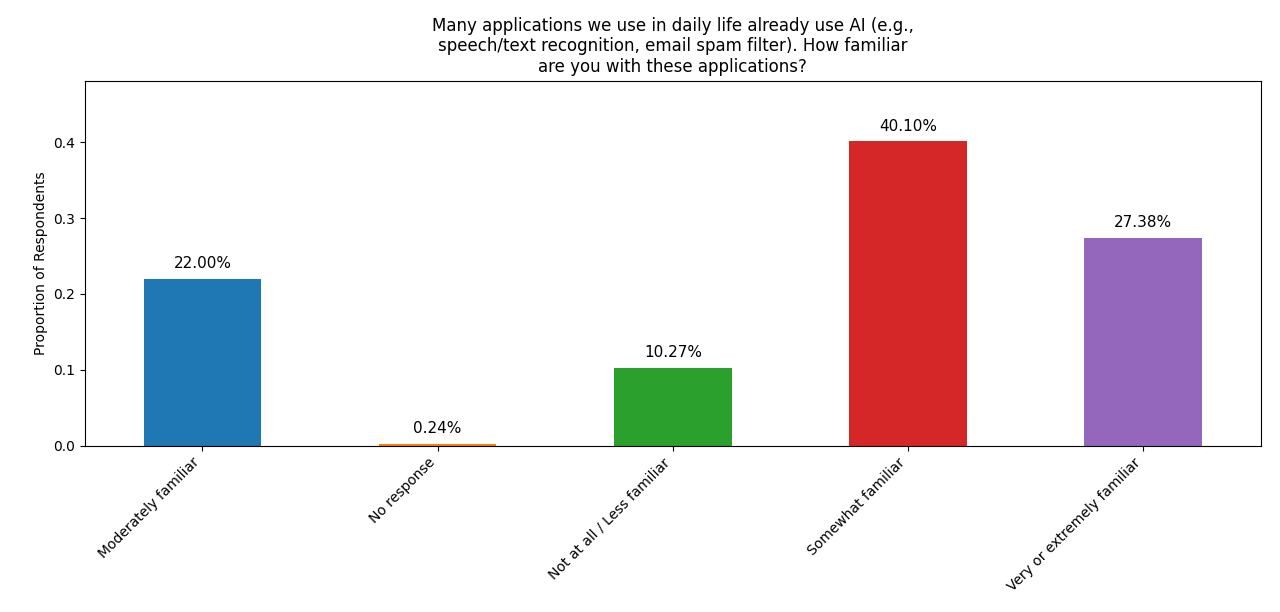


A12: Tech-savviness by gender.


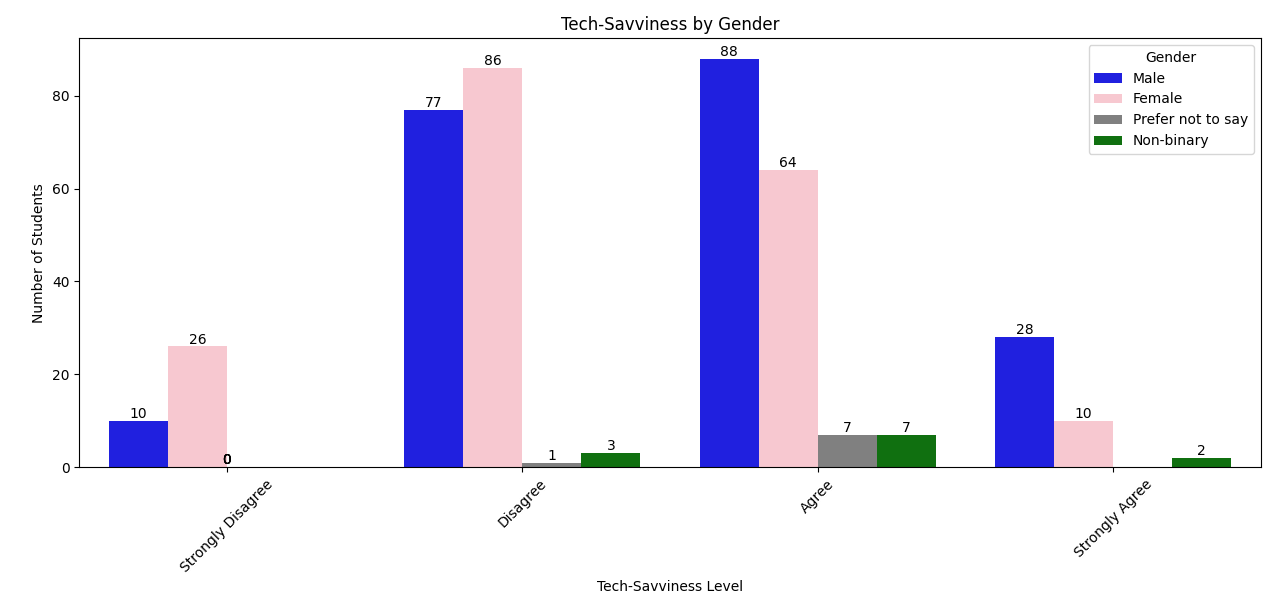


A13: Responses to “In the foreseeable future, all doctor will be replaced”


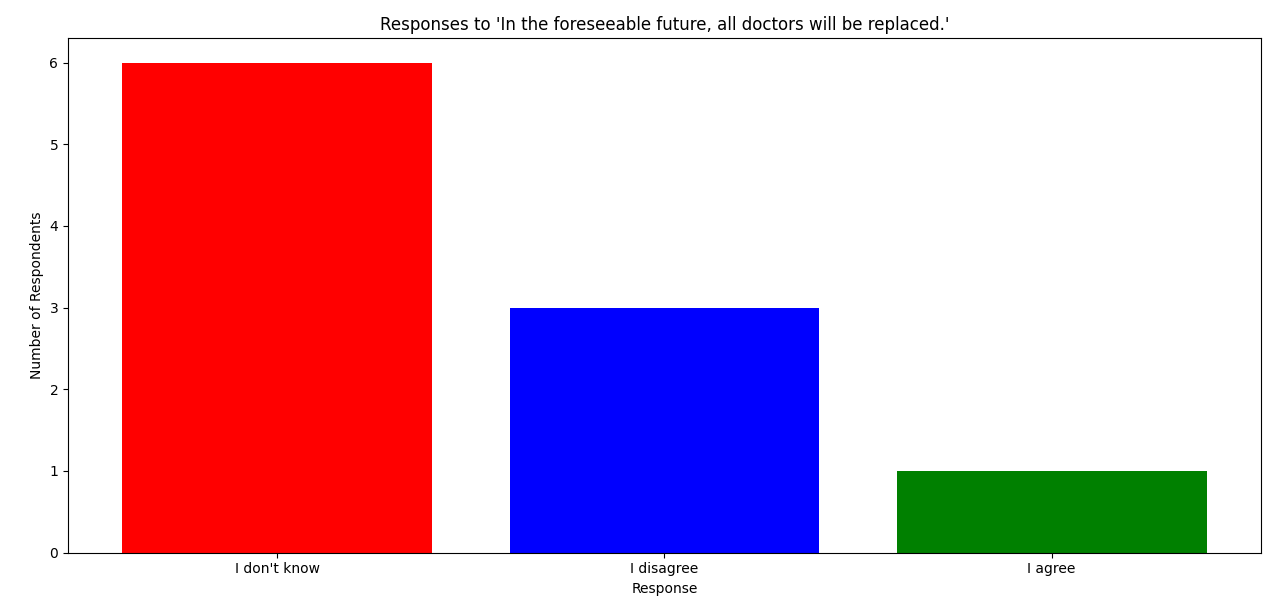


A.14: Distribution of fear regarding AI developments


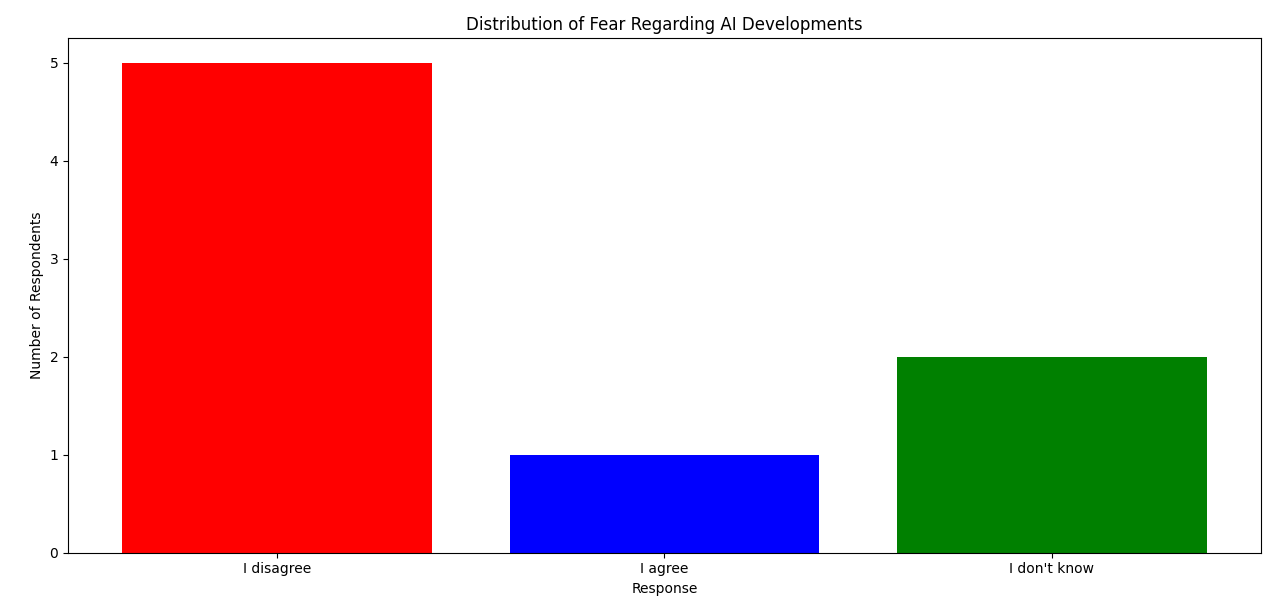


A.15: Distribution of enthusiasm about medical AI developments


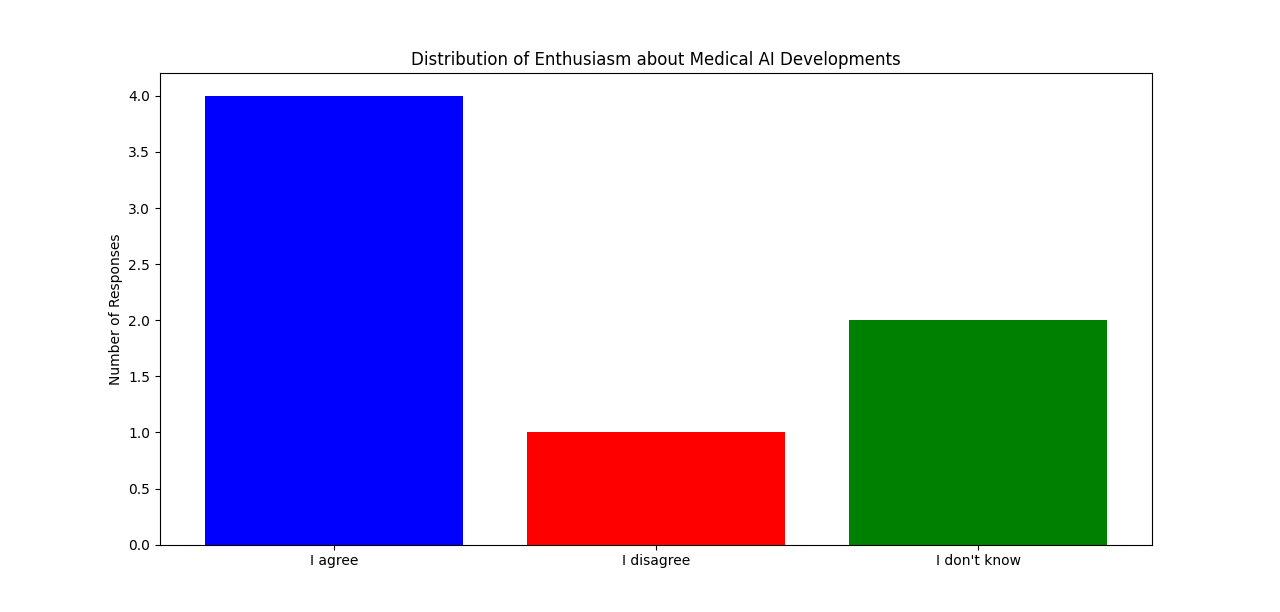


A.16: Distribution of AI Understanding by Country


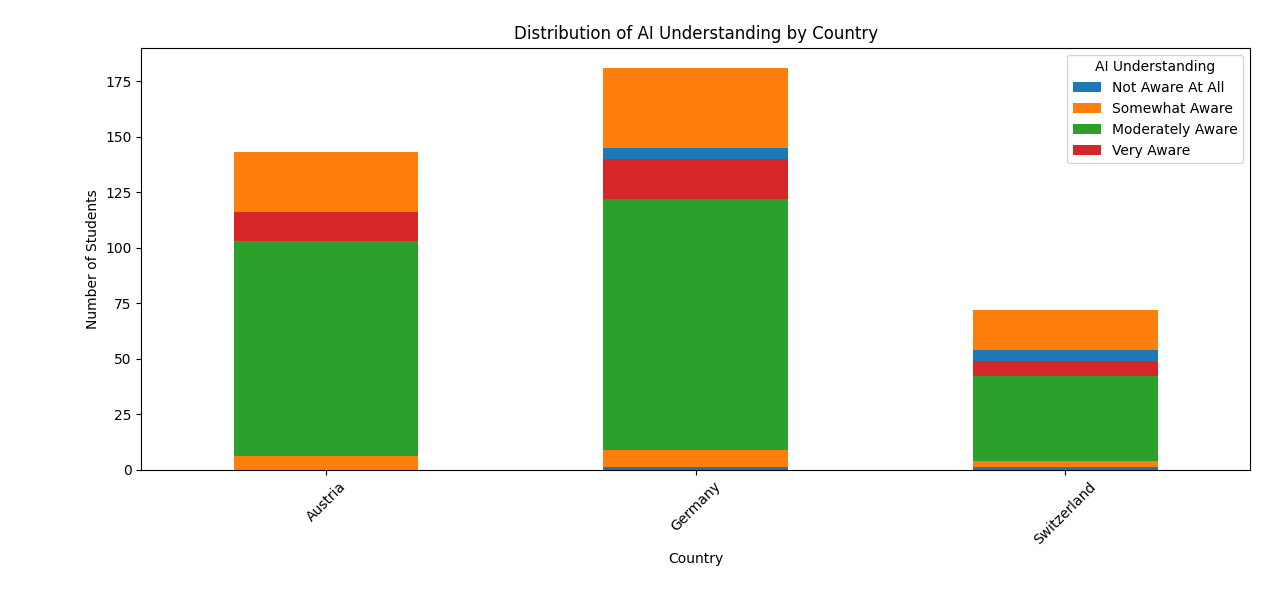


A 17: Tech-savviness among medical and dental students


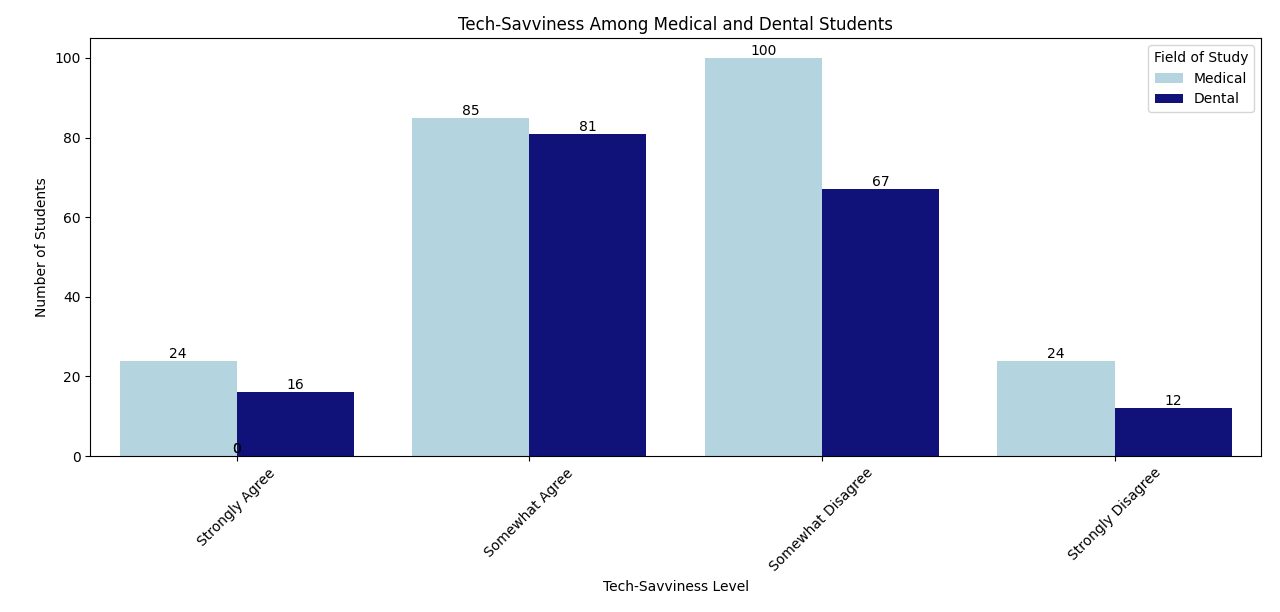


A 18: Distribution of AI Understanding by Gender


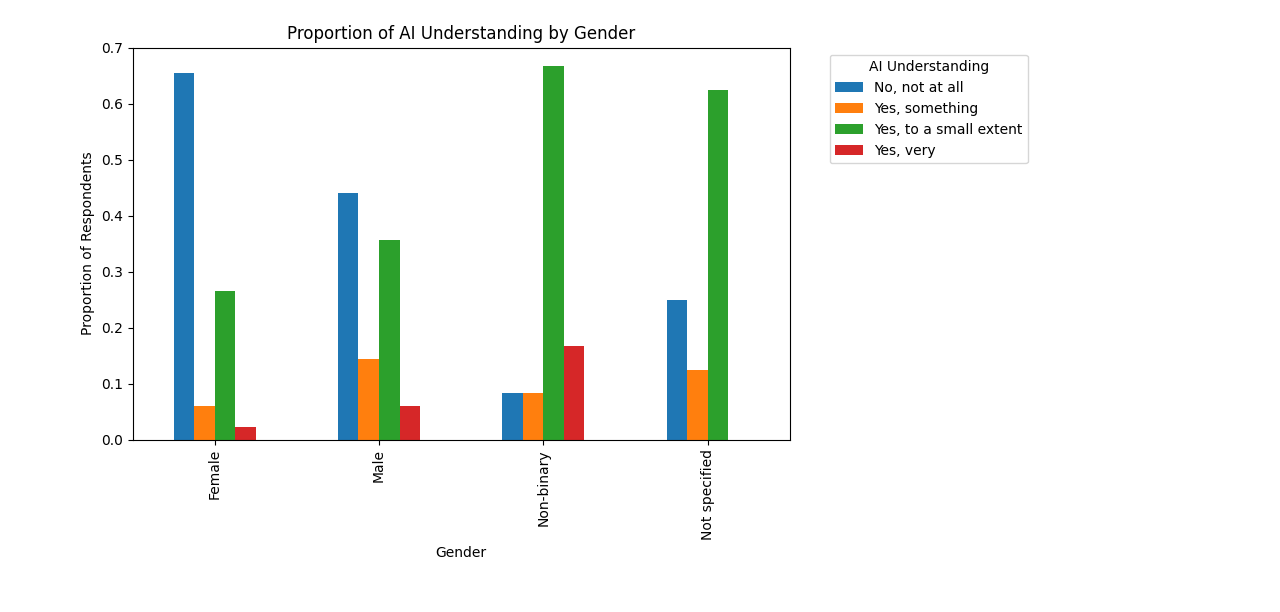


A 19: Distribution of AI Understanding by Country


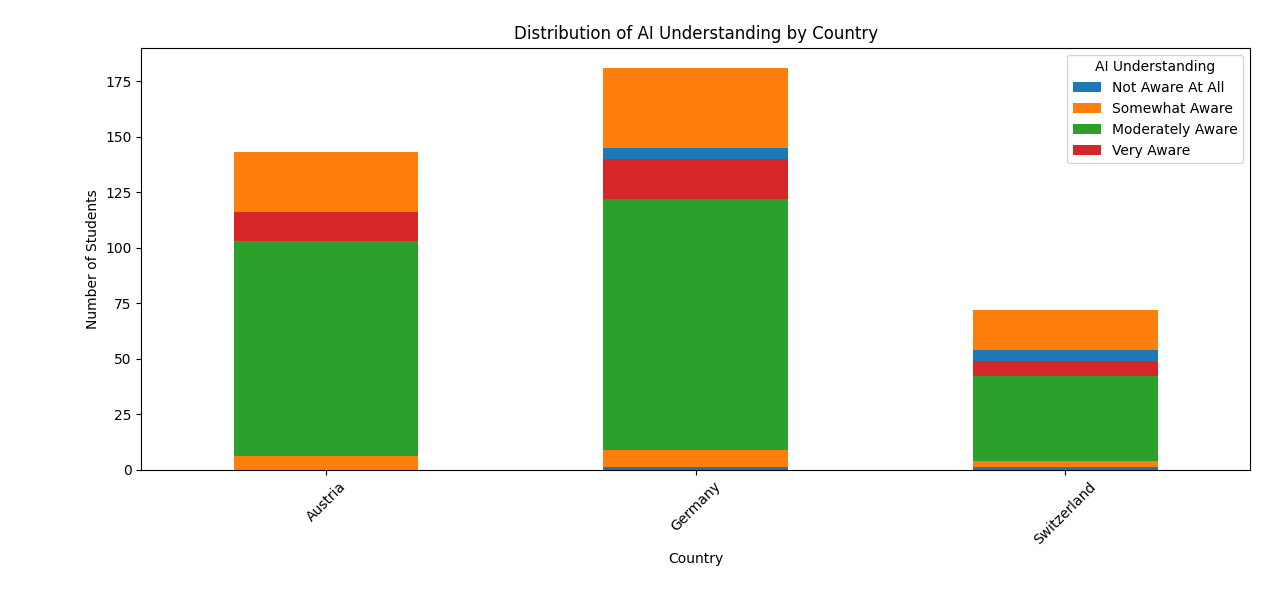


A.20: Mean Tech Affinity Scores for Non-AI trained and AI trained groups with Standard Deviation


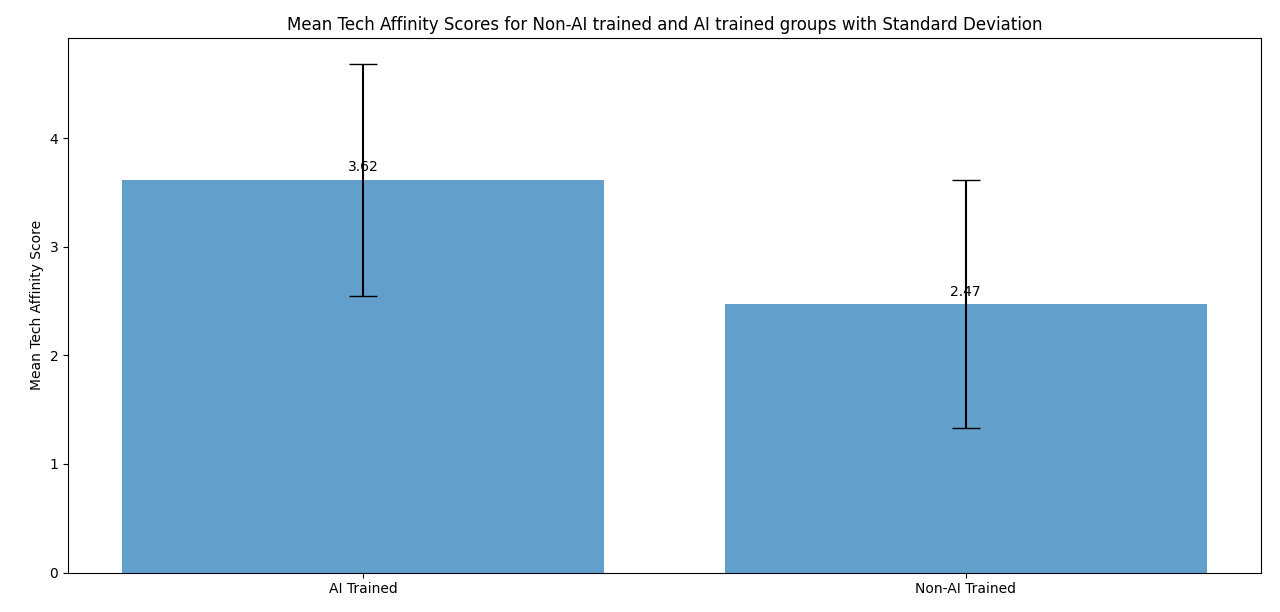


A.21: Online survey questionnaire (original on the left, translation on the right)

| **Abschnitt 1: Soziodemografische Daten** | **Section 1: Socio-demographic data** |
| --- | --- |
| 1. Wie alt sind Sie? | 1. How old are you? |
| 2. Was ist Ihr Geschlecht? | 2. What is your gender? |
| 3. Was studieren Sie? | 3. What are you studying? |
| 4. In welchem Studienabschnitt befinden Sie sich? | 4. In which stage of your studies are you? |
| 5. In welchem Studienjahr befinden Sie sich aktuell? | 5. In which academic year are you currently? |
| 6. In welchem Land studieren Sie? | 6. In which country are you studying? |
| 7. Inwieweit betrachten Sie sich selbst als technikaffin? | 7. To what extent do you consider yourself tech-savvy? |

| **Abschnitt 2: Verständnis der Grundprinzipien der KI** | **Section 2: Understanding the basic principles of AI** |
| --- | --- |
| 1. Künstliche Intelligenz (KI) ist ein Sammelbegriff für viele Technologien (z. B. 'Machine Learning'). Haben Sie ein grundlegendes Verständnis für diese Technologien? | 1. Artificial Intelligence (AI) is a collective term for many technologies (e.g., 'Machine Learning'). Do you have a basic understanding of these technologies? |
| 2. KI hat viele Anwendungen in der Medizin (z. B. KI-assistierte robotergestützte Chirurgie). Wie vertraut sind Sie mit diesen Anwendungen? | 2. AI has many applications in medicine (e.g., AI-assisted robotic surgery). How familiar are you with these applications? |
| 3. Viele Anwendungen, die wir im täglichen Leben anwenden, verwenden bereits KI (z. B. Sprach-/Texterkennung, E-Mail-Spam-Filter). Wie vertraut sind Sie mit diesen Anwendungen? | 3. Many applications we use in daily life already use AI (e.g., speech/text recognition, email spam filter). How familiar are you with these applications? |

| **Abschnitt 3: KI als Thema in Medizin und Zahnmedizin** | **Section 3: AI as a topic in medicine and dentistry** |
| --- | --- |
| 1. KI in der medizinischen Forschung entwickelt sich rasant. KI-Projekte in der Gesundheitsversorgung haben weltweit mehr Investitionen angezogen als KI-Projekte in jedem anderen Bereich. Wie bewusst sind Sie sich hierüber? | 1. AI in medical research is developing rapidly. AI projects in healthcare have attracted more investment worldwide than AI projects in any other field. How aware are you of this? |
| 2. ‘Künstliche Intelligenz’ und ‘DL’ werden derzeit in der medizinischen Gemeinschaft breit diskutiert. Wie bewusst sind Sie sich hierüber? | 2. Artificial Intelligence' and 'DL' are currently widely discussed in the medical community. How aware are you of this? |
| 3. Inwieweit haben Sie das Gefühl, dass Sie ein Verständnis für die Technologien haben, die der 'Künstlichen Intelligenz' und dem 'DL' zugrunde liegen? | 3. To what extent do you feel you have an understanding of the technologies that underpin 'Artificial Intelligence' and 'DL'? |
| 4. Haben Sie als Teil Ihrer (human-/zahn-)medizinischen Ausbildung eine Form von KI-Training erhalten, und wenn ja, um welche Art von Training handelte es sich? | 4. Have you received any form of AI training as part of your (human/dental) medical training, and if so, what type of training was it? |

| **Abschnitt 4: Thema des erhaltenen KI-Trainings** | **Section 4: Subject of the AI training you received** |
| --- | --- |
| 1. Geben Sie bitte das Thema des KI-Trainings an, das Sie erhalten haben: | 1. Please indicate the topic of the AI training you received: |

| **Abschnitt 5: Vertrauen in die Fähigkeit, vertrauenswürdige Informationen über KI in der Medizin/Zahnmedizin zu finden** | **Section 5: Trust in their ability to find trustworthy information about AI in medicine/dentistry** |
| --- | --- |
| 1. Wie sehr vertrauen Sie ihrer Fähigkeit, vertrauenswürdige Informationen über KI in der Medizin/Zahnmedizin zu finden? | 1. How much do you trust their ability to find trustworthy information about AI in medicine/dentistry? |

| **Abschnitt 6: Gründe für fehlendes Vertrauen angeben** | **Section 6: Give reasons for lack of trust** |
| --- | --- |
| 1. Geben Sie bitte die Gründe für Ihr fehlendes Vertrauen an: | 1. Please state the reasons for your lack of confidence: |

| **Abschnitt 7: Einstellungen und Gefühle gegenüber KI** | **Section 7: Attitudes and feelings towards AI** |
| --- | --- |
| 1. Ich betrachte KI in der Medizin als Partner und nicht als Konkurrenten. | 1. I consider AI in medicine as a partner and not as a competitor. |
| 2. Künstliche Intelligenz wird die Medizin/Zahnmedizin im Allgemeinen revolutionieren. | 2. Artificial intelligence will revolutionize medicine/dentistry in general. |
| 3. In absehbarer Zukunft werden alle Ärzte ersetzt werden. | 3. All doctors will be replaced in the foreseeable future. |
| 4. Diese Entwicklungen machen mir Angst. | 4) These developments scare me. |
| 5. Durch diese Entwicklungen erscheint mir die Medizin im Allgemeinen aufregender. | 5. these developments make medicine in general seem more exciting to me. |
| 6. KI wird den menschlichen Arzt niemals entbehrlich machen. | 6. AI will never make the human doctor dispensable. |
| 7. KI wird die Medizin im Allgemeinen verbessern. | 7. AI will improve medicine in general. |
| 8. KI sollte Teil der medizinischen/zahnärztlichen Ausbildung sein. | 8. AI should be part of medical/dental education. |

| **Abschnitt 9: Integration von KI in die Ausbildung** | **Section 9: Integration of AI into education** |
| --- | --- |
| 1. In welcher Form sollte KI-Teil der Ausbildung sein? | 1. In what form should AI be part of education? |
| 2. Wann sollte KI-Teil der Ausbildung sein? | 2. When should AI be part of education? |
| 3. Welche Inhalte sollte KI-Ausbildung umfassen? | 3. What content should AI training include? |

1. The higher education system in Germany, Austria and Switzerland consists of a tiered structure. The “Vorklinik/Bachelor” phase refers to the first 1-3 years of undergraduate medical or dental study typically focused on pre-clinical coursework. The “Klinik/Master” phase signifies advanced undergraduate study involving clinical rotations and practice. “Doktorat/PhD” refers to graduate level study and research for a doctoral degree. [↑](#footnote-ref-1)
